# On a Dhole Trail: Examining Ecological and Anthropogenic Correlates of Dhole Habitat Occupancy in the Western Ghats of India

**DOI:** 10.1371/journal.pone.0098803

**Published:** 2014-06-03

**Authors:** Arjun Srivathsa, Krithi K. Karanth, Devcharan Jathanna, N. Samba Kumar, K. Ullas Karanth

**Affiliations:** 1 Post-Graduate Programme in Wildlife Biology and Conservation, Wildlife Conservation Society, India Program, National Centre for Biological Sciences (Tata Institute of Fundamental Research), Bangalore, India; 2 Centre for Wildlife Studies, Bangalore, India; 3 Wildlife Conservation Society, Bronx, New York, United States of America; 4 Wildlife Conservation Society, India Program, Bangalore, India; 5 Nicholas School of Environment, Duke University, Durham, North Carolina, United States of America; Panthera United States of America

## Abstract

Although they play a critical role in shaping ecological communities, many threatened predator species are data-deficient. The Dhole *Cuon alpinus* is one such rare canid with a global population thought to be <2500 wild individuals. We assessed habitat occupancy patterns of dholes in the Western Ghats of Karnataka, India, to understand ecological and anthropogenic determinants of their distribution and habitat-use. We conducted spatially replicated detection/non-detection surveys of dhole signs along forest trails at two appropriate scales: the entire landscape and a single wildlife reserve. Landscape-scale habitat occupancy was assessed across 38,728 km^2^ surveying 206 grid cells of 188-km^2^ each. Finer scale habitat-use within 935 km^2^ Bandipur Reserve was studied surveying 92 grid cells of 13-km^2^ km each. We analyzed the resulting data of dhole signs using likelihood-based habitat occupancy models. The models explicitly addressed the problematic issue of imperfect detection of dhole signs during field surveys as well as potential spatial auto-correlation between sign detections made on adjacent trail segments. We show that traditional ‘presence versus absence’ analyses underestimated dhole habitat occupancy by 60% or 8682 km^2^ [naïve = 0.27; 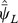
(SE) = 0.68 (0.08)] in the landscape. Addressing imperfect sign detections by estimating detection probabilities [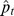 *_(L)_* (SE) = 0.12 (0.11)] was critical for reliable estimation. Similar underestimation occurred while estimating habitat-use probability at reserve-scale [naïve = 0.39; 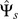
(SE) = 0.71 (0.06)]. At landscape scale, relative abundance of principal ungulate prey primarily influenced dhole habitat occupancy. Habitat-use within a reserve, however, was predominantly and negatively influenced by anthropogenic disturbance. Our results are the first rigorous assessment of dhole occupancy at multiple spatial scales with potential conservation value. The approach used in this study has potential utility for cost-effectively assessing spatial distribution and habitat-use in other species, landscapes and reserves.

## Introduction

Large carnivores are highly threatened across the world [1] and populations face risks of local extinction because they are wide-ranging and occur at relatively low densities [2]–[4]. Wide-ranging carnivores are often found across diverse habitats such as forests, open grasslands, cultivated lands and agricultural landscapes. Therefore, understanding carnivore distributions at the spatial scale of large landscapes is crucial for identifying sites for targeted conservation efforts [5]–[8]. Even within designated wildlife reserves, effective conservation requires data on how carnivores use different habitat types in a matrix. Furthermore, a full understanding of large-scale carnivore distribution as well as patterns of habitat-use within wildlife reserves can be gained only by identifying ecological and anthropogenic factors that are key determinants of habitat occupancy.

Wild canids, in particular, have large geographic ranges with interspersion of human-modified landscapes. Consequently, dependence on common prey resources has led to conflict, persecution by humans and spread of disease from domestic animals [9], [10]. The Asiatic Wild Dog or Dhole *Cuon alpinus*, Pallas 1811, is the only Asian wild canid that primarily inhabits forested areas. Dholes are among the top social predators of large ungulates in tropical forests [11]–[13]. Their numbers have significantly declined and trace populations are now largely restricted to forested areas [14]. In India, dholes were considered vermin and bounty-hunted to the verge of extinction before they received legal protection in 1972 [14], [15]. They have been extirpated from 60% of their former range in the last century due to human persecution and loss of forest cover, and now occur primarily in protected wildlife reserves embedded within larger multiple-use landscapes [16], [17].

Although historically a widespread species, current subjective assessments suggest that <2500 individuals of dholes may survive globally [14]. Despite their endangered status [14], dholes are the least studied social large carnivores. Previous studies of dholes have mostly focused on diet profiles [18]–[21] and behavior through *ad libitum* data collection methods [22]–[24]. This dearth of quantitative studies of their population ecology hinders conservation. Therefore, an assessment of dhole distribution and habitat-use is important in elucidating ecological drivers of their present occurrence and for measuring the viability of remaining populations and habitats.

A multi-scale analysis of spatial distribution is important for conservation in order to isolate scale-dependent ecological processes [25]. For example, assessment of landscape-level distribution of dholes could provide information on meta-population structure, population source-sites, functional corridors, landscape connectivity and other threats, which are useful for regional conservation planning. In areas where dholes are known to occur, such as individual wildlife reserves, patterns of habitat-use can provide insights on ecological drivers of local densities and efficacy of management interventions.

Because dhole signs such as tracks and scats are relatively easy to identify, we designed rigorous sign surveys under an occupancy-modeling framework. We examined dhole occupancy at two distinct spatial scales identified as relevant for understanding dhole ecology and addressing conservation needs. At landscape-scale, we surveyed a 38, 728 km^2^ area along the Western Ghats (Karnataka, India) to examine dhole distribution patterns. In order to assess patterns of fine-scale habitat-use, we chose a subset of the larger landscape, and surveyed a 935-km^2^ area in a single wildlife reserve.

Animal distribution patterns across space are typically non-uniform as a consequence of varying habitat characteristics [26], [27]. Variations in distribution patterns can be estimated when data from well-designed field surveys are confronted with ecologically relevant predictors and analyzed through parsimonious modeling [28]. Factors such as abundance of prey species, land cover type, anthropogenic disturbances and protection efforts may influence spatial distribution of carnivores [29]–[31]. We hypothesized that for dholes, site-specific probabilities of occupancy at both scales would be influenced by a combination of ecological and anthropogenic factors.

Prey densities are fundamental determinants of carnivore densities [32], [33]. We measured relative abundances of all prey species (gaur *Bos gaurus*, sambar *Rusa unicolor*, chital *Axis axis*, wild pig *Sus scrofa* and muntjac *Muntiacus muntjak*) that make up >90% of biomass in dhole diet [18], [19], [23], [24]. However, our interest was in identifying the influence of preferred prey species abundance on distribution of dholes. Based on previous diet studies, we predicted that chital and sambar abundance would positively influence dhole occupancy at both spatial scales. However, anecdotal records also indicate that dholes avoid human settlements and presence. We therefore also predicted that anthropogenic disturbance would negatively influence dhole distribution and habitat-use.

This is the first systematic application of robust occupancy models to quantitatively assess dhole distribution and habitat-use patterns anywhere in their range.

## Materials and Methods

### Ethics Statement

The study was conducted in the protected areas and adjoining forests of Western Ghats within the state of Karnataka. The Karnataka State Forest Department provided necessary research permits for the study. Since the methods used were non-invasive and relied completely on recording indirect signs of animals, animal care and use committee approval was not required.

### Study Area

**Figure 1:**
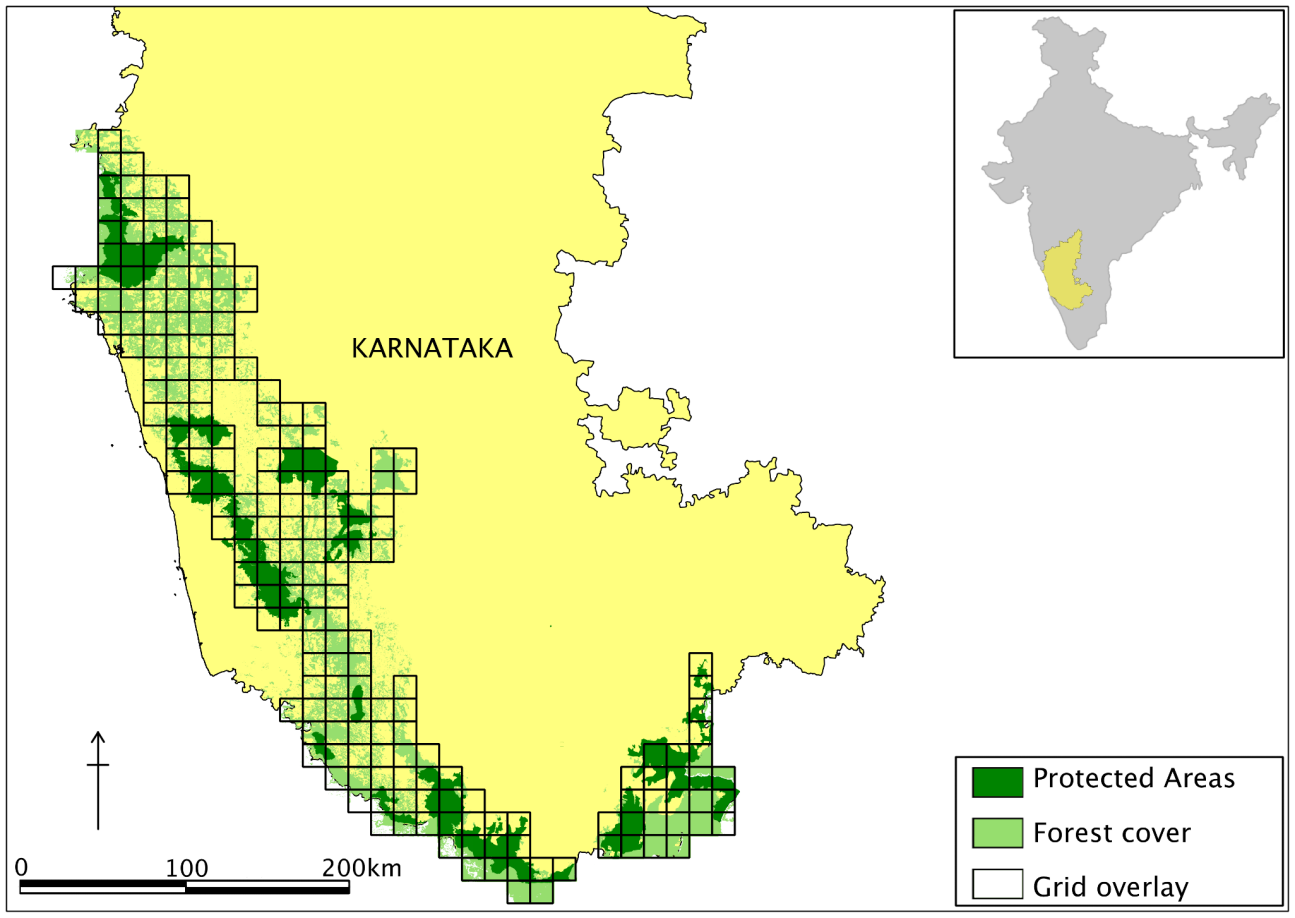
Study area map of the Western Ghats landscape in Karnataka. Study area and survey design used for the landscape scale habitat occupancy of dholes in the Western Ghats, Karnataka State, India (2006–2007). The map shows overall forest cover, protected wildlife reserves with superimposition of 188 km^2^-grid-array. Inset: location of the study area in India.

A countrywide occupancy-based questionnaire survey of large mammal distributions in India showed dhole presence in the Western Ghats, Central India and North-east India [16]. Our study was conducted in the central part of the Western Ghats, located in the state of Karnataka (Fig.1).

We assessed landscape-scale dhole occupancy across a 38,728 km^2^ area in Karnataka's Western Ghats. The study area consists of semi-evergreen, tropical moist-deciduous, tropical dry-deciduous and dry-deciduous forests with substantial anthropogenic modifications, creating a heterogeneous vegetation matrix [34]. There are 16 protected reserves in the landscape, encompassing an area of *c*. 8700 km^2^. The 21,176 km^2^ of forested areas support a diverse assemblage of wild ungulates such as the chital, four-horned antelope *Tertracerus quadricornis*, gaur, mouse deer *Moschiola indica*, muntjac, sambar and wild pig. The landscape also has populations of dhole and its co-predators, the tiger *Panthera tigris* and the leopard *Panthera pardus* [33].

**Figure 2:**
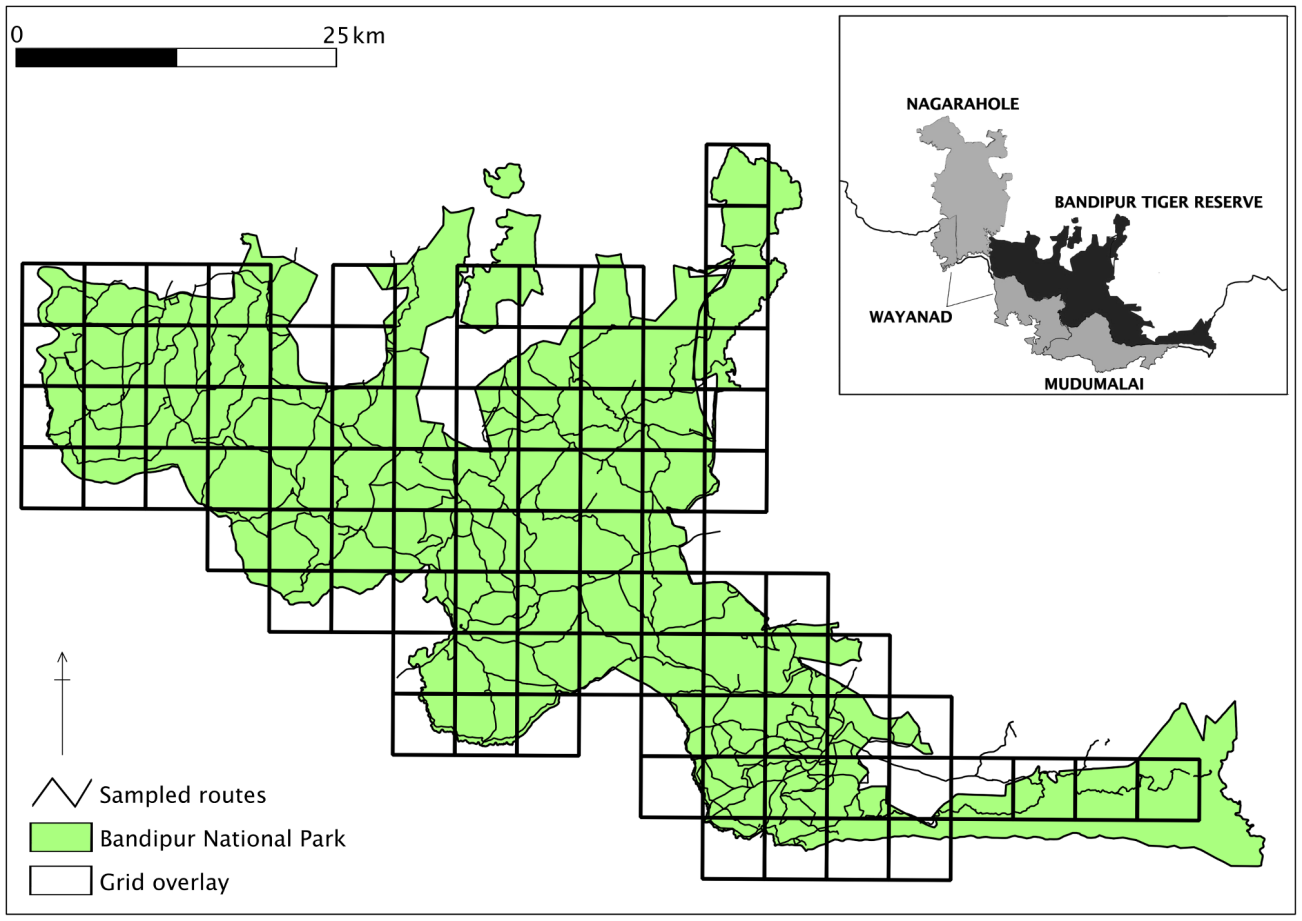
Study area map of Bandipur Tiger Reserve. Study area and survey design for Bandipur Tiger Reserve, India (2012) showing protected area boundary, forest road sign-survey routes and 13-km^2^-grid array. Inset: location of the study area and adjoining protected areas.

To examine fine-scale habitat-use by dholes, we chose Bandipur National Park, a 935-km^2^ subset of the Western Ghats landscape in Karnataka. This protected area predominantly has tropical moist and dry deciduous forests, with some areas degraded to scrub due to human impacts [35]. It supports a density of 35.2/100 km^2^ medium to large sized prey species [33]. It is contiguous with Wayanad Wildlife Sanctuary and Mudumalai Wildlife Sanctuary to its south and Nagarahole National Park to its northwestern sides (Fig. 2).

### Occupancy Modeling

Although conventional radio-telemetry can generate reliable data on large carnivore habitat-use, its application is limited because it is expensive, labor-intensive and involves the difficult tasks of safe capture, immobilization and handling of elusive animals. Due to these difficulties, emphasis has increasingly shifted to collection of non-invasive data such as indirect signs, camera-trap photographs, fecal DNA, etc. to estimate distribution and abundances [36]–[39].

From such data, estimates of space-use at the scale of patch or habitat are sometimes obtained using Resource Selection Functions (RSF) or Resource Selection Probability Functions (RSPF). Weaknesses of the approach include the need for some estimate of resource ‘availability’ which is often difficult to obtain in practice [48]. Moreover, since such functions do not take variable detectability into account, it usually results in imprecise estimates and flawed inferences on species-habitat relationships [40], [41]. Other ‘predictive’ distribution approaches such a Habitat Suitability Models (HSM) and Ecological Niche Factor Analysis [42] that rely on presence-only data introduce biases in estimates because of imperfect detection issues, unequal sampling effort and unaccounted true absence of species [43], [44].

On the other hand, occupancy modeling permits using detection/non-detection data of animal signs, while explicitly addressing the issue of imperfect detection [26], [28], [45]. This method works well for studies of carnivores that generally occur at low local densities and large spatial scales [31], [46], [47]. Recent occupancy models can also yield reliable estimates of probability of habitat-use at finer spatial scales [48]–[50]. The stronger inference derived from occupancy models arises from their ability to make full use of available information, decomposing true absence from non-detection, within a probabilistic framework [48], [51], [52].

### Field Survey Methods

Dholes use forest roads and trails extensively for hunting, movement, scent-marking and defecation at latrine sites [18], [19], [23]. Therefore, surveying for their signs such as tracks and scat-piles along roads/trails improves detection rates and sample sizes. To optimize spatial coverage and logistical feasibility, we implemented occupancy survey design using spatial rather than temporal replicates, surveying consecutive segments along forest roads and trails [53], [54]. A team of three surveyors skilled in mammal sign recognition surveyed forest roads and trails to detect signs of dholes. Only fresh, correctly identifiable signs were recorded to avoid biases arising from misclassifications or sign decay [55], [56].

#### Landscape-scale survey design

We adapted the occupancy sampling design implemented for surveys of tigers in the same landscape [31], [54]. Field surveys were carried out between February 2006–May 2006 and December 2006–May 2007 (during the dry season) maintaining uniform detection conditions with an assumption that dhole distribution did not change over the larger landscape in this short period.

The study area was gridded with 206 square cells of 188-km^2^ size each to estimate true habitat occupancy (proportion of area occupied by dholes). Since we aimed at estimating the proportion of total area occupied by dholes at large scale, it was necessary that each grid (sample unit) was larger than the maximum home range size of dhole packs in the area. We relied on home range sizes estimated from sighting records of known individual packs in earlier studies [23], [24] as well as radio-telemetry based estimates from other landscapes [57], setting cell sizes much larger than these estimates (range 20–105 km^2^). We standardized the sampling effort per cell such that walk effort was ∼40 km for a cell that was fully occupied by dhole habitat. The effort reduced in proportion to available dhole habitat as measured by extent of forest cover.

Detections of dhole signs were recorded as either ‘1’ (detected) or ‘0’ (undetected) on each successive 100-meter long trail/road segment. For analyses these detection/non-detection data were later aggregated at 1-km segments. The number of spatial replicates we sampled per cell varied from 2 to 42. The survey effort invested was 2021 man-days with 4174 km distance walked, yielding a total of 278 detections of dhole signs (211 scat deposit locations and 67 track sets). In terms of the number of replicates, as well as signs detected, the survey generated data appropriate for formal occupancy analyses.

#### Reserve-scale survey design

Within the larger landscape, at the scale of a single reserve field surveys were conducted during the dry season (January 2012–April 2012) in Bandipur Tiger Reserve. A grid with 92 cells of 13 km^2^ each was overlaid on the area. The estimated dhole home-range size of *c.* 20–40 km^2^ in the dry season [23], [24] was larger than the cell size with the objective being measuring patterns of ‘habitat-use probability’ rather than proportion of habitat occupied (true habitat occupancy). Our attempt was to maximize spatial coverage and to discriminate between more and less intensively used areas by dhole within a single reserve. Therefore, the entire network of dirt roads within Bandipur was sampled intensively (Fig. 2). Detection/non-detection data were recorded from consecutive 100-meter segments along forest-roads. The number of replicates per cell varied from 8 to 254 100-m segments. We surveyed 92 such small cells with a total of 730 km of walk effort, resulting in 235 detections of dhole signs (57 fresh scat deposits and 178 track sets).

### Analytical Approach

The standard occupancy model of MacKenzie et al. [28] describes the likelihood of observing a detection history for a site (detections and non-detections) as a function of the occurrence probability (occupancy, Ψ) and detection probability (detectability, *p*). Maximum likelihood methods may be used to estimate these parameters [58].

We used an extension to the standard model above, developed by Hines et al. [54] that uses spatial replicates instead of temporal ones, and explicitly accounts for spatial auto-correlation of detections on contiguous replicates. This model has been applied successfully for surveys of other carnivores (see Karanth et al. [31] and Sunarto et al. [59] for tigers; Thorn et al. [60] for brown hyaenas *Hyaena brunnea*). The key parameters estimated by the Hines et al. [54] spatial dependence model are:

***Ψ*** - probability of dhole presence in a site

***θ^0^*** - probability of dhole presence in a replicate conditional on absence in the previous replicate

***θ^1^*** - probability of dhole presence in a replicate conditional on presence in the previous replicate

***p_t_*** - probability of detecting dhole sign in a replicate conditional on presence in the replicate

The detection parameters ***θ^0^*** and ***θ^1^*** express the magnitude of spatial dependence between contiguous replicates (***θ^0^*** = ***θ^1^*** signifies complete independence of replicates). We use the subscripts ‘L’ and ‘s’ with each of these parameters to indicate two spatial scales. Ψ*_L_* refers to probability of dhole occurrence at landscape-scale and Ψ*_s_* refers to probability of habitat-use by dholes at reserve-scale.

### Covariates for distribution and habitat-use

Detections of signs of principal ungulate prey species were recorded on spatial replicates within each sampled grid cell. Because direct observations of anthropogenic disturbances are rare and difficult to quantify, we used indirect evidence, such as signs of livestock presence (e.g. tracks, dung, pellets, etc.) as reasonable surrogates of human disturbance factors (see Karanth et al. [31]). These covariates were quantified as a ratio of number replicates with indirect signs to the total number of replicates in each site.

We modeled site-specific probabilities of dhole occupancy Ψ*_i_* as linear functions of the above mentioned covariates using a logit link function [28], [40]:
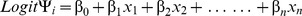
where *β_i_* refers to the magnitude of influence of individual ecological or anthropogenic covariate *x_i_*. All the site-specific covariates measured as proportions were converted to scaled and centered values [61]. We used model selection tests based on Akaike information criterion (AIC) implemented in program PRESENCE version 5.7 [62] to rank competing covariate models [63].

## Results

### Occupancy models to address spatial auto-correlation

When estimating occupancy at both landscape and reserve scales, we first compared the standard MacKenzie et al. [28] model with the Hines et al. [54] model that explicitly addresses the likely spatial auto-correlation of sign detections made along spatial replicates. These models fit the data better based on AIC values, than the standard model that assumes such sign detections are independent events (Table 1). Parameter estimates showed lack of independence strongly at both scales (Table 2).

**Table 1:** Comparison between the standard occupancy model [28] and the model using spatial replicates with potential auto-correlation of sign detections among them [54] used to generate estimates of dhole habitat occupancy at landscape scale (Western Ghats, India, 2006–2007) and habitat-use at reserve scale (Bandipur Tiger Reserve, India, 2012).

		AIC	ΔAIC	AIC weight	Model Likelihood	K*	Deviance
	Model						
**Landscape-scale occupancy**	*Ψ_L_ (.) θ^0^_L_ (.) θ^1^_L_ (.) p_t(L)_ (.)*	975.47	0	1	1	5	965.47
	*Ψ_L_ (.) p_(L)_ (.)*	1005.91	30.44	0	0	3	999.91
**Reserve-scale occupancy**	*Ψ_s_ (.) θ^0^_s_ (.) θ^1^_s_ (.) p_t(s)_ (.)*	991.27	0	1	1	5	981.27
	*Ψ_s_ (.) p_s_ (.)*	1508.67	517.4	0	0	3	1502.67

*K =  number of parameters.

**Table 2:** Estimates of dhole habitat occupancy at landscape-scale (Western Ghats, India, 2006–2007) and habitat-use at reserve-scale (Bandipur) generated using spatially replicated sign surveys under the model [54] incorporating spatial auto-correlation of sign detections.

	Naïve	*Ψ (SE)*	*θ^0^ (SE)*	*θ^1^ (SE)*	*p_t_ (SE)*
**Landscape-scale occupancy**	0.27	0.75 (0.32)	0.03 (0.09)	0.88 (0.04)	0.19 (0.03)
**Reserve-scale occupancy**	0.39	0.71 (0.12)	0.01 (0.00)	0.75 (0.04)	0.78 (0.06)

Please see Methods section for parameter descriptions.

Footnote: Here the parameters Ψ and *p_t_* are estimated only for the basic model with no covariates. Final estimates of these parameters were derived from covariate models.

### Landscape-scale Occupancy

#### Detection probability of dhole signs

We initially modeled the replicate-level detection probability (*p_t(L)_*) as a function of abundance of preferred prey (chital, sambar) and human disturbance (livestock presence) as plausible ecological and anthropogenic covariates, while maintaining a global model for the occupancy parameter [*Ψ_L_*(*chital+sambar+livestock*)]. We estimated cell-specific detection probability *p_t(L)_* at landscape-scale as a function of combined abundance of all prey species (*chital+sambar+gaur+pig+muntjac*). We also used human disturbance (livestock sign abundance) as an additive variable expected to negatively influence dhole occupancy. The best fit model (AIC weight  = 0.60) showed that *p_t(L)_* was a function of combined abundance of all prey. We used this model for all subsequent analyses (Table 3).

**Table 3:** Results of comparisons to select models for estimating probability of detecting dhole signs *p_t_* on 1-km long spatial replicates used in the field survey conducted at landscape scale in the Western Ghats landscape, India (2006–2007), under the constant global model for dhole occurrence [*Ψ_L_* (*chital+sambar+livestock)*].

	AIC	ΔAIC	AIC weight	Model Likelihood	K*	Deviance
Model						
*Ψ_L_ (global) θ^0^_L_ (.) θ^1^_L_ (.) p_t(L)_ (allprey)*	966.33	0	0.6031	1	6	954.33
*Ψ_L_ (global) θ^0^_L_ (.) θ^1^_L_(.) p_t(L)_(allprey+livestock)*	967.21	0.88	0.3884	0.644	7	953.21
*Ψ_L_ (global) θ^0^_L_ (.) θ^1^_L_ (.) p_t(L)_ (.)*	975.47	9.14	0.0062	0.0104	5	965.47
*Ψ_L_ (global) θ^0^_L_ (.) θ^1^_L_ (.) p_t(L)_ (livestock)*	977.47	11.14	0.0023	0.0038	6	965.47

*K = number of parameters.

#### Probability of occurrence

We tested six models with different sets of plausible covariates against the basic model [here the basic model refers to Ψ*_L_ (.), θ^0^_L_ (.), θ^1^_L_ (.), p_t(L)_ (allprey)*]. We used Akaike model weights to assess to strength of evidence in favor of each of these seven models (Table 4). Four models that include abundance of chital, sambar as positive influences and livestock signs as negative variables ranked higher in the candidate set (ΔAIC<2.0). We derived the maximum likelihood parameter estimates for occupancy at landscape-scale (Ψ*_L_*) from models with Akaike weight >0.01 using model averaging [63].

**Table 4:** Model comparisons to identify ecological and anthropogenic habitat covariates influencing dhole distribution Ψ*_L_* at landscape-scale (Western Ghats, India, 2006–2007) from spatially replicated sign surveys during 2006–2007.

	AIC	ΔAIC	AIC weight	Model Likelihood	K*	Deviance
Model						
*Ψ_L_ (chital+livestock) θ^0^_L_ (.) θ^1^_L_ (.) p_t(L)_ (allprey)*	961.98	0	0.2958	1	8	945.98
*Ψ_L_ (chital) θ^0^_L_ (.) θ^1^_L_ (.) p_t(L)_ (allprey)*	962.02	0.04	0.2899	0.9802	7	948.02
*Ψ_L_ (chital+sambar) θ^0^_L_ (.) θ^1^_L_ (.) p_t(L)_ (allprey)*	962.89	0.91	0.1876	0.6344	8	946.89
*Ψ_L_ (chital+sambar+livestock) θ^0^_L_ (.) θ^1^_L_ (.) p_t(L)_ (allprey)*	963.62	1.64	0.1303	0.4404	9	945.62
*Ψ_L_ (.) θ^0^_L_ (.) θ^1^_L_ (.) p_t(L)_ (allprey)*	966.33	4.35	0.0336	0.1136	6	954.33
*Ψ_L_ (livestock) θ^0^_L_ (.) θ^1^_L_ (.) p_t(L)_ (allprey)*	966.44	4.46	0.0318	0.1075	7	952.44
*Ψ_L_ (sambar) θ^0^_L_ (.) θ^1^_L_ (.) p_t(L)_ (allprey)*	966.49	4.51	0.031	0.1049	7	952.49

*K = number of parameters.

#### Influence of covariates on dhole distribution

We had predicted that variations in distribution at the landscape-scale would be a function of ecological and anthropogenic variables. We further hypothesized that abundance of wild ungulate prey would positively influence dhole distribution, whereas human disturbance (indicated by livestock presence) would have a negative influence. Therefore, we examined the untransformed β coefficient values of these variables. The magnitude and direction (sign) on the β coefficient values for chital and sambar were positive, as we had expected (Table 5).

**Table 5:** Estimates of β coefficient values for different individual habitat covariates hypothesized to influence dhole distribution Ψ*_L_* at landscape scale (Western Ghats, India, 2006–2007).

	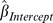 *(SE)*	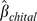 *(SE)*	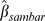 *(SE)*	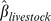 *(SE)*
Model				
*Ψ_L_ (chital+livestock) θ^0^_L_ (.) θ^1^_L_ (.) p_t(L)_ (allprey)*	1.38 (1.11)	4.52 (3.01)	-	−1.18 (0.94)
*Ψ_L_ (chital) θ^0^_L_ (.) θ^1^_L_ (.) p_t(L)_ (allprey)*	1.26 (1.04)	4.05 (2.81)	-	-
*Ψ_L_ (chital+sambar) θ^0^_L_ (.) θ^1^_L_ (.) p_t(L)_ (allprey)*	1.57 (1.30)	4.84 (3.41)	0.99 (1.29)	-
*Ψ_L_ (chital+sambar+livestock) θ^0^_L_ (.) θ^1^_L_ (.) p_t(L)_ (allprey)*	1.50 (1.17)	4.85 (3.20)	0.56 (1.06)	−1.00 (0.94)
*Ψ_L_ (.) θ^0^_L_ (.) θ^1^_L_ (.) p_t(L)_ (allprey)*	1.21 (1.46)	-	-	-
*Ψ_L_ (livestock) θ^0^_L_ (.) θ^1^_L_ (.) p_t(L)_ (allprey)*	1.50 (2.11)	-	-	−1.63 (2.30)
*Ψ_L_ (sambar) θ^0^_L_ (.) θ^1^_L_ (.) p_t(L)_ (allprey)*	2.27 (4.11)	-	4.47 (9.48)	-
***Model averaged values***	**1.42 (1.28)**	**4.05 (2.8)**	**0.40 (0.73)**	**−0.53 (0.56)**

These include model-averaged β estimates with unconditional standard errors.

Human disturbance negatively influenced dhole distributions as indicated by the negative sign on β values. The model-specific β coefficients for all covariates at landscape-scale, along with model-averaged values are presented in Table 5. We summed the Akaike weights of key covariates in order to examine the relative influence of each covariate. Chital abundance was found to have the highest influence on landscape-scale occupancy (summed Akaike weight  = 0.90).

#### Parameter estimates of dhole distribution

The final parameter estimates of dhole occupancy and replicate-level detection probabilities were derived from model averaging across the covariate models. The model-averaged estimate of probability of landscape-scale occupancy was 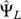 (SE) = 0.67 (0.08).

We used the mean estimate of probability of occupancy at large scale to arrive at total proportion of the landscape occupied by dholes (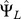). For this, the cell-specific values of occupancy were obtained as a weighted product of probability of occupancy (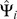) and proportional area of forest cover in each cell (*a_i_*/21,176).
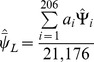



**Figure 3:**
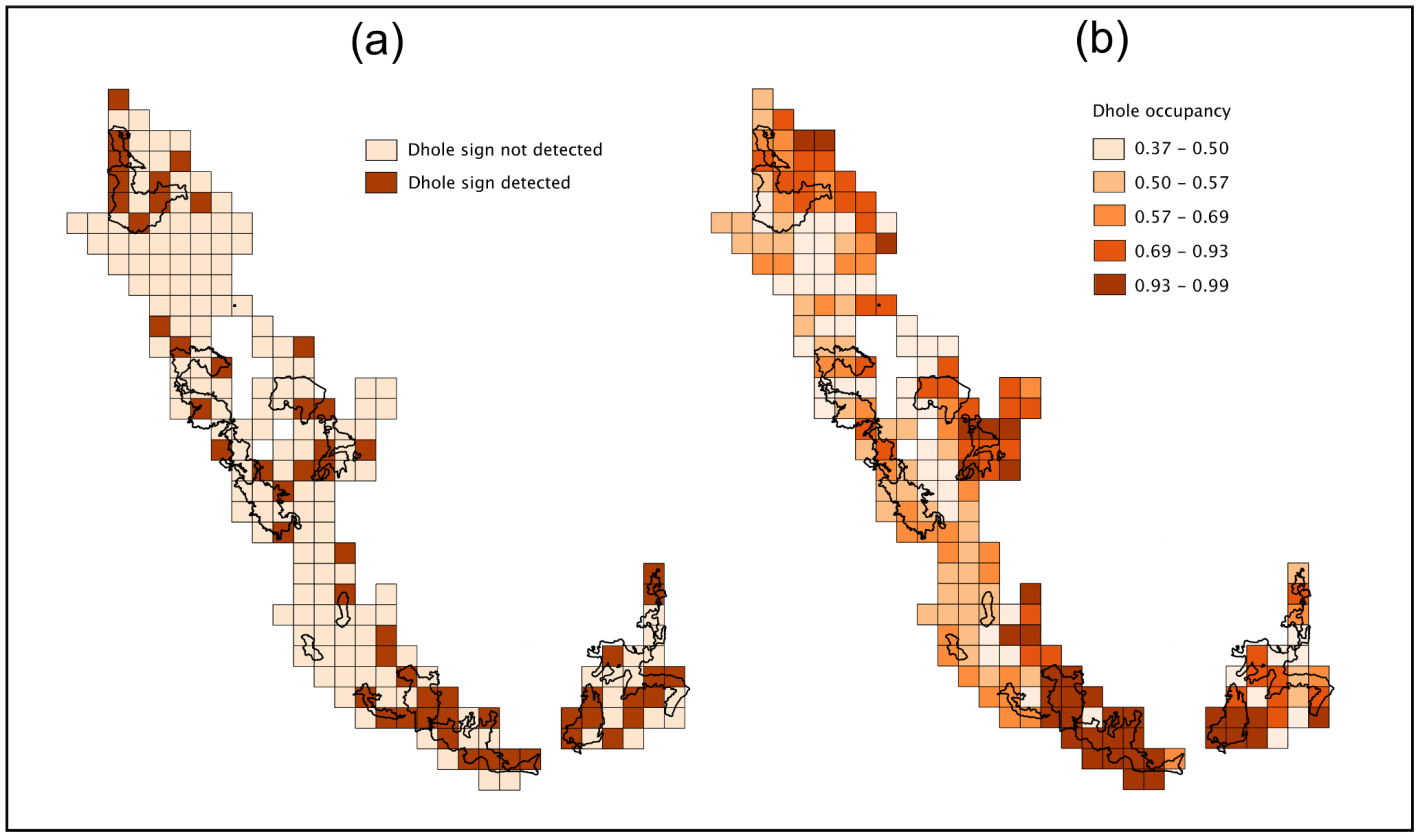
Dhole distribution patterns. Patterns of landscape-scale occupancy: dhole distribution in the Western Ghats of Karnataka, India (2006–2007). (a) Naïve estimate and (b) estimated probabilities of occupancy.

The conventional presence-absence approach indicated dhole occupancy in the Western Ghats landscape to be 27% (*c.* 5,700 km^2^). After accounting for imperfect detection [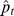 *_(L)_*(SE) = 0.12 (0.11)], Markovian dependence of replicates, and model–averaging the occupancy parameter, we estimate that dholes occupied 14,185 km^2^ (68%) of the landscape [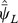
(SE) = 0.68 (0.08)], which shows that the presence-versus-absence approach severely underestimated true occupancy (Fig. 3).

### Reserve-scale Occupancy

#### Detection probability of dhole signs

At the reserve-scale, replicate level detectability was estimated from intensive surveys of the entire road-route network of Bandipur. This yielded a substantially high estimate of detection probability [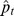 *_(s)_* (SE) = 0.78 (0.06)] even for the basic model [Ψ*_s_ (.), θ^0^_s_ (.), θ^1^_s_ (.), p_t(s)_ (.)*]. Therefore we used constant detectability *p_t(s)_ (.)* in modeling probability of habitat-use (Ψ*_s_*).

#### Probability of habitat-use

We chose seven plausible covariate models and tested them against the basic model [here, the basic model refers to Ψ*_s_ (.), θ^0^_s_ (.), θ^1^_s_ (.), p_t(s)_ (.)*] (Table 6). Highly cross-correlated variables were not included in the same model (for example: Pearson correlation coefficient ***r_chital_***_** x *sambar***_ = 0.52). We note that at this scale, the basic model without any covariates showed the best fit. However, the difference among AIC values across all models was low (ΔAIC<2.52) with all competing models having Akaike weights >0.01. Therefore, at this scale also we performed model averaging to estimate occupancy, defined as probability of habitat-use (Ψ*_s_*) within the reserve.

**Table 6:** Model comparisons to identify ecological and anthropogenic habitat covariates influencing dhole habitat-use Ψ*_s_* at reserve scale (Bandipur Tiger Reserve, India) from spatially replicated sign surveys during January-April 2012.

	AIC	ΔAIC	AIC weight	Model Likelihood	K*	Deviance
Model						
*Ψ_s_ (.) θ^0^_s_ (.) θ^1^_s_ (.) p_t(s)_ (.)*	991.27	0	0.2406	1	5	981.27
*Ψ_s_ (livestock) θ^0^_s_ (.) θ^1^_s_ (.) p_t(s)_ (.)*	991.81	0.54	0.1837	0.7634	6	979.81
*Ψ_s_ (sambar) θ^0^_s_ (.) θ^1^_s_ (.) p_t(s)_ (.)*	992.20	0.93	0.1511	0.6281	6	980.20
*Ψ_s_ (sambar+livestock) θ^0^_s_ (.) θ^1^_s_ (.) p_t(s)_ (.)*	993.02	1.75	0.1003	0.4169	7	979.02
*Ψ_s_ (allprey) θ^0^_s_ (.) θ^1^_s_ (.) p_t(s)_ (.)*	993.12	1.85	0.0954	0.3965	6	981.12
*Ψ_s_ (chital) θ^0^_s_ (.) θ^1^_s_ (.) p_t(s)_ (.)*	993.23	1.96	0.0903	0.3753	6	981.23
*Ψ_s_ (allprey+livestock) θ^0^_s_ (.) θ^1^_s_ (.) p_t(s)_ (.)*	993.73	2.46	0.0703	0.2923	7	979.73
*Ψ_s_ (chital+livestock) θ^0^_s_ (.) θ^1^_s_ (.) p_t(s)_ (.)*	993.79	2.52	0.0682	0.2837	7	979.79

*K = number of parameters.

#### Influence of covariates on dhole habitat-use

We had hypothesized that even at the reserve scale, abundance of preferred ungulate prey species would positively influence dhole habitat-use, and human disturbance would be a negative influence. Therefore, we examined the untransformed β coefficient values of these variables. At this scale, however, only sambar abundance positively influenced dhole habitat-use but chital abundance as well as that of all prey combined had little effect.

Human disturbance negatively influenced probability of habitat-use as shown by the negative sign on β values. The model-specific β coefficients for covariates at reserve-scale, along with model-averaged values are in Table 7. As in the case of the large-scale, we summed the Akaike weights of key variables in order to examine the relative influence of each covariate. Dhole habitat-use pattern within the reserve was most significantly influenced by human disturbance (summed Akaike weight  = 0.42).

**Table 7:** Estimates of β coefficient values for different individual habitat covariates hypothesized to influence dhole habitat-use Ψ*_s_* at reserve scale (Bandipur Tiger Reserve, India, 2012).

	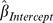 *(SE)*	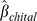 *(SE)*	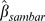 *(SE)*	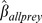 *(SE)*	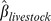 *(SE)*
Model					
*Ψ_s_ (.) θ^0^_s_ (.) θ^1^_s_ (.) p_t(s)_ (.)*	0.88 (0.57)	-	-	-	-
*Ψ_s_ (livestock) θ^0^_s_ (.) θ^1^_s_ (.) p_t(s)_ (.)*	0.85 (0.58)	-	-	-	−0.96 (0.76)
*Ψ_s_ (sambar) θ^0^_s_ (.) θ^1^_s_ (.) p_t(s)_ (.)*	1.11 (0.89)	-	1.35 (1.83)	-	-
*Ψ_s_ (sambar+livestock) θ^0^_s_ (.) θ^1^_s_ (.) p_t(s)_ (.)*	1.00 (0.75)	-	1.09 (1.53)	-	−0.93 (0.78)
*Ψ_s_ (allprey) θ^0^_s_ (.) θ^1^_s_ (.) p_t(s)_ (.)*	0.92 (0.61)	-	-	0.29 (0.78)	-
*Ψ_s_ (chital) θ^0^_s_ (.) θ^1^_s_ (.) p_t(s)_ (.)*	0.90 (0.60)	0.17 (0.92)	-	-	-
*Ψ_s_ (allprey+livestock) θ^0^_s_ (.) θ^1^_s_ (.) p_t(s)_ (.)*	0.88 (0.62)	-	-	0.22 (0.80)	−0.94 (0.76)
*Ψ_s_ (chital+livestock) θ^0^_s_ (.) θ^1^_s_ (.) p_t(s)_ (.)*	0.89 (0.61)	0.12 (0.93)	-	-	−0.96 (0.76)
***Model averaged values***	**0.96 (0.68)**	**0.02 (0.15)**	**0.32 (0.50)**	**0.05 (0.16)**	**−0.43 (0.42)**

These include model-averaged β estimates with unconditional standard errors.

#### Parameter estimates of dhole habitat-use

**Figure 4:**
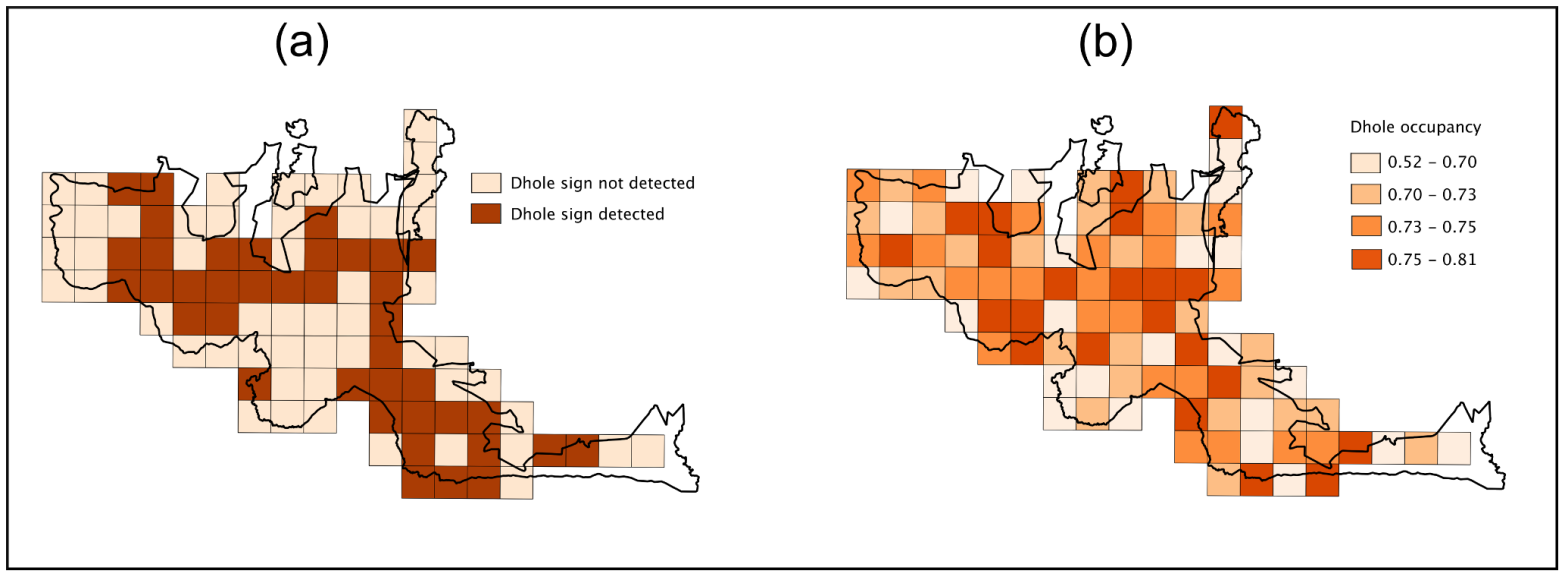
Dhole habitat-use patterns. Patterns of reserve-scale occupancy: habitat-use by dholes in Bandipur Tiger Reserve, India (2012). (a) Naïve estimate from presence-versus-absence approach and (b) estimated probabilities of occupancy.

The naïve ‘presence-versus-absence’ estimate suggested that dholes used about 39% of Bandipur National Park. We estimated true probability of use to be 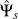 (SE) = 0.71 (0.06) and detectability 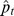 *_(s)_* (SE) = 0.78 (0.06). Site-specific variations in Ψ*_s_* calculated from averaging across all covariate models, show a matrix of high and low habitat-use probabilities (Fig. 4). These interspersed high and low habitat-use patches suggest that even within an occupied wildlife reserve, dholes selectively use certain habitats more than others.

## Discussion

### Methodological Issues

Animal sign surveys are efficient because they do not require technically trained field personnel or advanced equipment. Consequently, they have wide applicability in assessing animal distributions at large spatial scales [28], [37]. Surveys using an occupancy modeling framework can estimate and predict species distributions at single population or meta-population levels, scaling up from individual reserves to larger landscapes or regions.

In our study, as expected, a traditional presence-versus-absence approach underestimated dhole occupancy by 8682 km^2^ (60%). Analysis under the approach used by Hines et al. [54] and Karanth et al. [31] for surveying tigers overcame this bias and generated a reasonable estimate of landscape occupancy at 68% (14,185 km^2^). The models we used partitioned the observation process [52] into components of detection probability at the replicate level and cell level, thus fully addressing the twin problems of imperfect detection as well as autocorrelation of sign detections (Table 2). The superiority of this approach was clearly demonstrated at both reserve scale and landscape scale, with the models clearly fitting the data better (Tables 1 and 2). We believe this approach is superior to other predictive distribution assessments that use only data on presence [44], [64], [75], because it uses information built into absence data that is traditionally discarded. Furthermore, our study adapted the temporal replication-based model to one that is based on spatial replications, which is logistically more efficient in practice [31].

Although absolute abundance is a more sensitive metric for managing endangered wildlife populations, this parameter is difficult and expensive to derive at large spatial scales [51], [65]. Since dholes do not have distinct morphological traits (body/pelage markings), generating simultaneous estimates of distributions at large scales and habitat-use patterns at reserve scale may be of great value because abundance estimation methods that rely on individual identification from photographic captures [66] cannot be used. In the future, we anticipate development of models that can integrate landscape-scale occupancy data with reserve-scale dhole abundances derived using potentially useful methods such as genetic capture-recapture sampling [38]. While we have tried to reduce biases in our estimates from misclassification of indirect signs (incorrect identification of tracks and scats from other wild carnivores or feral/domestic dogs), we do not discount the possibility of potential errors. Recent developments in occupancy modeling have addressed these issues [55], though currently there are no methods that integrate spatial dependence models [54] with false-positive error models.

### Ecological interactions, habitat characteristics and dhole occupancy

The interactions between a species and its habitat(s) may occur at multiple scales [67]. Our results reaffirm the importance of choosing the appropriate spatial scale for assessing species-habitat relationships. At the landscape scale, surveys of tigers that used cells (sites) much larger than expected home range size yielded reliable inferences on true occupancy [31], [54] and possibly even relative abundance [68]. In our study, at the landscape-scale we could reliably estimate proportion of potential habitat occupied by dholes. The influence of ecological and anthropogenic covariates at the cell (site) level on dhole distribution was generally in conformity with our predictions (Table 4). Abundance of chital emerged as the most important ecological driver in determining dhole distribution. However, distribution of chital in the landscape was limited to deciduous forests with flatter terrain [69, this study]. Wherever chital were absent, sambar appears to be principal prey species influencing dhole occurrence (Table 5).

‘Probability of occupancy’ (using large cells) is not a very useful metric at the level of individual reserves where dholes are present. The parameter ‘probability of habitat-use’, which measures intensity with which dholes use different areas within a reserve (Bandipur) is more useful for identifying important habitats for dholes. Estimates of probability of habitat-use in our study showed how factors influencing species-habitat relationships are scale dependent. For example, chital abundance, a key covariate in determining distribution of dholes at landscape scale, does not appear to affect habitat-use patterns of dholes at reserve scale, as does sambar abundance (Table 7). We note that chital occur in large herds (up to 81 animals/group) compared to sambar (up to 6 animals/group) [70]. Because chital occur in almost every site (13 km^2^ cell in our study), the relationship between their abundance and habitat occupancy breaks down at smaller scales [71]–[73]. Chital attain high densities in all surveyed cells within Bandipur [74] and therefore their abundance does not appear to influence fine-scale habitat-use by dholes. We further examined this issue *post hoc* by including combined abundance of all ungulate prey as a covariate in the reserve-scale model comparisons (Table 6). The combined prey abundance also had no influence on patterns of habitat-use by dholes (Table 7), confirming that cell-specific abundance of select prey species is a strictly scale-dependent factor in determining dhole occupancy. On the other hand, anthropogenic disturbance (typified by livestock presence in our study) clearly and negatively influences dhole distribution at landscape scale (Table 5) as well as dhole habitat-use at the reserve scale (Table 7).

Carnivore densities and other demographic parameters are chiefly influenced by prey abundance [32], [33], [75]–[77]. Our results highlight the importance of medium to large-sized prey for driving dhole occupancy across the landscape, in concordance with other studies of dhole feeding ecology in the Western Ghats and across Asia [12], [13], [19], [20], [78].

### Conservation Implications

Landscape-scale estimates of dhole occupancy presented in this paper show that dholes are not strictly confined to protected wildlife reserves as is generally perceived (Fig. 3). Dholes occupy about 14,185 km^2^ (68%) of the Karnataka Western Ghats landscape, of which only *c*. 8700 km^2^ (41%) is in protected wildlife reserves.

A meta-analysis by Woodroffe and Ginsberg [79] predicted 723 km^2^ as the lower reserve size threshold for persistence of dhole populations in India. With the average reserve size being 570 km^2^ in our study area, dhole populations seem to thrive in smaller reserves or even outside the reserve system, in multi-use forests. This is possibly enabled by larger populations in reserves like Bandipur-Nagarahole that serve as ‘sources’ in sustaining a dhole meta-population in the Western Ghats landscape of Karnataka. Meta-populations comprising of multiple small populations of animals are more likely to persist in landscapes with multiple high quality habitat patches with good connectivity [80] as in the Western Ghats. Therefore, retention of landscape connectivity beyond well-protected wildlife reserves should receive greater attention in this region.

Unlike tigers and leopards in this landscape, dholes are not involved in significant levels of conflict with humans [81] or targeted for illegal trade of body parts [82]. However, their persistence outside protected reserves is problematic, because of depleted prey densities [16], [33] and potential risk of disease from large populations of semi-feral dogs and cats [83]. The Indian government is currently promoting voluntary village relocations from protected wildlife habitats as a strategy for conflict mitigation and improving social welfare [84], [85]. This could be a very effective indirect tool for ensuring dhole population viability. However, with rapid economic growth in the Western Ghats, many infrastructure projects involving highways, pipelines, dams, canals and power lines are likely to increase habitat fragmentation and subsequently impact dhole populations. We believe that our results may be very useful to mitigate the impact of such projects, if considered seriously while planning regional infrastructure development. Voluntary relocation projects and establishment of ecologically sensitive zones [86] may also benefit from considering spatial distribution patterns of dhole populations reported in this study.

At the level of a single reserve (Bandipur), the available prey abundance appears to be more than adequate to support current densities of dholes [33], [74]. Abundance index of chital and combined prey species measured in our study show presence in almost all grids with very high relative abundance across grids. Current management practices include habitat manipulations in the form of creating water holes and artificially increasing forage availability to increase herbivore populations. Such targeted habitat manipulations to further increase prey abundance, particularly that of chital which are at excessive densities (>50–100 animals/km^2^) in some locations [74], therefore seem unnecessary. In view of the significant negative influence of human disturbance on dhole occupancy, in the landscape as well as within the designated wildlife reserve, conservation resources should be focused on strengthening protection and patrolling to reduce such impacts.

With major declines in carnivore numbers globally and the data-deficient status of most dhole populations [1], [3], our findings have potential utility for conservation of dholes across their geographic range. While we reliably and rigorously identified key ecological drivers of dhole occupancy at multiple spatial scales, we believe our approach has wider application for assessing distribution and habitat-use patterns in other rare, elusive and wide-ranging carnivore species.
