# Correction: On a Dhole Trail: Examining Ecological and Anthropogenic Correlates of Dhole Habitat Occupancy in the Western Ghats of India

**DOI:** 10.1371/journal.pone.0106213

**Published:** 2014-08-15

**Authors:** The PLOS ONE Staff

K. Ullas Karanth is incorrectly listed as being affiliated with the Nicholas School of Environment, Duke University, Durham, North Carolina, United States of America, in place of Krithi K Karanth, who is affiliated with the Nicholas School of Environment, Duke University. The publisher apologizes for this error.

The correct affiliation is: Arjun Srivathsa^1,2*^, Krithi K. Karanth^2,3,5^, Devcharan Jathanna^2^, N. Samba Kumar^2,4^, K. Ullas Karanth^2,3^

1 Post-Graduate Programme in Wildlife Biology and Conservation, Wildlife Conservation Society, India Program, National Centre for Biological Sciences (Tata Institute of Fundamental Research), Bangalore, India,

2 Centre for Wildlife Studies, Bangalore, India,

3 Wildlife Conservation Society, Bronx, New York, United States of America,

4 Wildlife Conservation Society, India Program, Bangalore, India,

5 Nicholas School of Environment, Duke University, Durham, North Carolina, United States of America

## References

[1] Srivathsa A, Karanth KK, Jathanna D, Kumar NS, Karanth KU (2014) On a Dhole Trail: Examining Ecological and Anthropogenic Correlates of Dhole Habitat Occupancy in the Western Ghats of India. PLoS ONE 9(6): e98803 doi:10.1371/journal.pone.0098803 24893166 10.1371/journal.pone.0098803 PMC4043888

